# Does palliative chemotherapy really palliate and are we measuring it correctly? A mixed methods longitudinal study of health related quality of life in advanced soft tissue sarcoma

**DOI:** 10.1371/journal.pone.0210731

**Published:** 2019-09-26

**Authors:** Nicholas Gough, Jonathan Koffman, Joy R. Ross, Julia Riley, Ian Judson

**Affiliations:** 1 Palliative Care Department, Guy’s and St Thomas’s Hospital NHS Foundation Trust, London, England, United Kingdom; 2 The Institute of Cancer Research, London, England, United Kingdom; 3 Kings College London, Cicely Saunders Institute, Department of Palliative Care, Policy and Rehabilitation, London, England, United Kingdom; 4 Royal Marsden and Royal Brompton Palliative Care service, Royal Marsden NHS Foundation Trust, London, England, United Kingdom; 5 National Heart and Lung Institute, Imperial College, London, England, United Kingdom; 6 Sarcoma Unit, Royal Marsden NHS Foundation Trust, London, England, United Kingdom; University of Texas M. D. Anderson Cancer Center, UNITED STATES

## Abstract

**Objective:**

Soft tissue sarcoma (STS) is a rare cancer type that when locally advanced or metastatic, is predominantly treated with palliative chemotherapy with the aim of improving both quantity and quality of life. Given modest survival data after commencing first line chemotherapy, this study examines (i) what constitutes health related quality of life (HRQoL), (ii) whether the most commonly used HRQoL assessment tool measures this and (iii) to what extent HRQoL, and its components, change during and after treatment.

**Design:**

Mixed-methods longitudinal study of 66 sarcoma patients living with STS (42 commencing chemotherapy, 24 under surveillance after completing chemotherapy) involving serial EORTC QLQ-C30 questionnaires and nested-qualitative semi-structured interviews with a sub-sample of participants. EORTC QLQ-C30 score change from baseline to primary evaluation point was examined using a paired t-test. Interviews were analysed using the framework approach before both datasets were integrated.

**Results:**

Five main factors, including control of pain, were identified by study participants as important components of HRQoL; these are examined within the EORTC QLQ-C30. However, others e.g. independence loss and common causes of anxiety, are not. Whilst social and psychological domains are addressed by the EORTC QLQ-C30, the quantitative change over time did reflect qualitative descriptions of decline.

The mean overall EORTC QLQ-C30 HRQoL score deteriorated from baseline (60.4) to the primary evaluation point (50.2) [change of -10.2, t-test: -2.70, p = 0.01] for those receiving chemotherapy; this was in concordance with patients’ qualitative accounts. Baseline overall HRQoL scores were higher in the surveillance group suggesting a correlation with chemotherapy response and longer-term improvement in HRQoL. The evidence from both HRQoL scores and qualitative accounts indicated that the presence and control of physical symptoms were particularly important in maintaining HRQoL. Whilst fatigue deteriorated on chemotherapy (baseline 41.7 to 52.8; change of +11.1, t-test +2.51, p<0.05), pain (baseline 41.5 to 32.1; change -9.4, t-test -2.06 p<0.05) and sleep disturbance (43.1 to 28.5; change -14.6, t-test –3.05, p<0.05) both improved.

**Conclusion:**

A key finding was that the EORTC QLQ-C30 assesses some but not all of the patient-reported components of HRQoL in sarcoma patients highlighting the need for either STS specific modules within the EORTC QLQ-C30 or a completely new STS specific HRQoL tool. First line palliative chemotherapy improves specific symptoms known to be prevalent and to influence HRQoL in this patient group which in some patients may translate to sustained improvement in HRQoL: further exploration and validation of these findings in larger prospective studies are warranted.

## Introduction

Soft tissue sarcomas are a heterogeneous group of rare malignant tumours accounting for 1% of all adult cancers with an incidence of approximately 5 per 100,000 per year[1,2]. Surgery, often supplemented by adjuvant radiotherapy, offers the only reliable chance of cure for localised disease.[3] However, over 50% of patients will develop metastases and die of their disease [4]. In the locally advanced inoperable or metastatic setting, systemic chemotherapy is the mainstay of treatment [4,5]; its principal aim is to palliate i.e. establish disease control in order to improve both quantity and quality of life.

Chemotherapy may also be used in the adjuvant or neoadjuvant setting. Although one of the largest adjuvant studies to be conducted to date was negative [6], recent data from the Italian Sarcoma Group lend weight to the view that neo-adjuvant chemotherapy may improve progression-free (PFS) and overall survival (OS) in selected groups of patients at high risk of relapse [7]. In the Italian study, standard chemotherapy with ifosfamide and epirubicin proved superior to non-anthracycline containing regimens in most of the chosen histotypes. Data are awaited from longer follow-up of this study. Since not all patients benefit from this approach, future studies of adjuvant and neo-adjuvant therapy should be performed incorporating HRQoL investigations.

Doxorubicin, alone or in combination with ifosfamide or olaratumab, remains standard first line palliative treatment for the majority of STS subtypes [8,9,10,11] Positive radiological response (Complete response [CR]/ partial response [PR]) rates to first line chemotherapy are modest, i.e. 10–40% [10,12] and median OS from commencing chemotherapy is 12–18 months [13,14,15]. Disease stabilisation (SD) is also an indication of treatment benefit in this disease, since the impact on survival is similar to that of PR, hence progression-free rate has been used as an end-point in phase II trials in sarcoma when determining whether or not to take a drug into a comparative phase III trial [16]. Treatment recommendations are influenced by the presence of symptoms, particularly pain and those caused by the mass effect of a space-occupying lesion [5,17] which chemotherapy aims to improve. Given the modest OS data and potential for chemotherapy related toxicity [18], there is a need to better understand and prospectively measure quality of life (QoL) in those with advanced STS to assess whether systemic anti-cancer treatments are providing effective palliation[19]. Moreover, there is now agreed consensus that assessment of QoL should be included alongside more traditional endpoints such as PFS and OS in all oncological drug trials [20].

### Health related quality of life and soft tissue sarcoma

The term health related quality of life (HRQoL) was developed to focus attention on how disease and its treatment affect individual well-being and physical function [21]. Whilst subjective and individualised in nature [22], it is accepted that overall HRQoL comprises physical, mental and psycho-social components[23]. A number of patient reported outcome questionnaires have been developed and validated in an attempt to measure this. Cancer-type specific HRQoL questionnaires exist for most common cancers [http://groups.eortc.be/qol/] but none have been developed specifically for patients with STS. Health related quality of life has been measured in studies of systemic therapy in STS using generic cancer HRQoL tools e.g. the EORTC QLQ-C30. However, it is not known whether what it measures is specific or relevant to those with advanced STS: qualitative techniques are needed to further explore this but to date there are no published qualitative studies in this cohort.

The aims of this study were two-fold: first, to explore qualitatively the individual constituents of HRQoL in two groups of patients with advanced STS: one group commencing first line palliative chemotherapy, the other undergoing a period of ‘active surveillance’ having completed, and favourably responded to, first line chemotherapy achieving at least stable disease. This allowed us to explore what aspects of HRQoL were important to these patients and whether the EORTC QLQ-C30 measured them. Second, we examined how overall HRQoL and its components changed over time in both groups using the EORTC QLQ-C30 and qualitative methodologies, to explore in what ways palliative chemotherapy was valuable in terms of HRQoL.

## Materials and methods

### Study Design

The study employed a longitudinal mixed methods study design to address the study aim. Specifically, mixed methods research represents a research design (or methodology) where both quantitative and qualitative approaches collect, analyse and integrate data into a single study [24].

Our rationale for this design was using both quantitative and qualitative components and asking distinct but intersecting questions would identify new dimensions of the complexity of HRQoL specific to STS than a single method alone [25,26,27] Specifically, we made use of quantitative assessments of HRQoL at three set time points using established, validated tools, and ‘nested’ qualitative interviews to explore patient participants’ perceptions of their illness and treatment experience.

### Study setting

We recruited patients with advanced STS attending the Royal Marsden (RM) NHS Foundation Trust between March 2011 and October 2012. The Royal Marsden sarcoma unit is one of the largest in the UK treating approximately 1000 new referrals a year.

### Study participants

#### Inclusion criteria

Participants were English-speaking adults and able to give informed consent. They had histologically confirmed, locally advanced, ‘inoperable’ or metastatic STS and were either (i) undergoing first-line palliative chemotherapy or (ii) under radiological surveillance after completion of first-line palliative chemotherapy having responded favourably: achieving at least stable disease. The choice of chemotherapy, i.e. doxorubicin, doxorubicin + ifosfamide or gemcitabine with or without docetaxel, was determined by a number of different factors including performance status, patient age and histology. These patients were recruited before the results of the GeDDiS trial were known, which demonstrated no advantage for gemcitabine plus docetaxel compared with doxorubicin, since when patients have not been treated with this combination first line[28]; and olaratumab had not yet become available.

#### Exclusion criteria

Those with low grade advanced STS (generally not treated with chemotherapy) or highly chemo-sensitive STS subtypes, for example, Ewing sarcoma that are potentially curable despite advanced presentation. The justification for excluding these patients is that in spite of the high toxicity of treatment there is currently no controversy regarding their management, i.e. no question as to whether or not treatment is justified. In contrast, positive radiological response rates (CR / PR or SD) to standard chemotherapy options for most STS are only in the region of 20–25% [10,11]. Consequently, the majority of patients experience the side-effects of chemotherapy in the absence of any objective evidence of benefit, even if disease stabilization is included. This may be due to a reduction in symptoms. Gastrointestinal stromal tumour (GIST) was excluded since this can be treated effectively with imatinib and other tyrosine kinase inhibitors that have a completely different set of associated side effects and are generally less toxic than chemotherapy.

### Recruitment and informed consent

Both in and out-patients who met the inclusion criteria were identified by the STS clinical team, introduced to NG and given a patient information sheet (PIS). After a minimum of 24-hours potential participants were re-contacted by NG to further explain the study and address any questions. In order to eliminate any potential for coercion, potential participants who declined to participate in the study were reassured verbally and in writing that their current /or future treatment and care would not be compromised in any way. Those who agreed to take part completed written informed consent to participate in the quantitative component (serial HRQoL questionnaires) and at the same time were provisionally asked if they would be willing to take part in a face to face semi-structured interview (the qualitative component) at a later date to further explore their ongoing STS and treatment experience. Consecutive patients agreeing to the qualitative component provided separate informed consent at the time of the face-to-face interview. Purposive sampling was used to give maximum variation to the sample by gender, age and treatment modality. Patients were reassured they could withdraw from either study component at any point in time. If participation to either component was declined, NG would politely ask patients to explain their reasons for doing.

### Data collection time points and timing of interview

**Fig 1:**
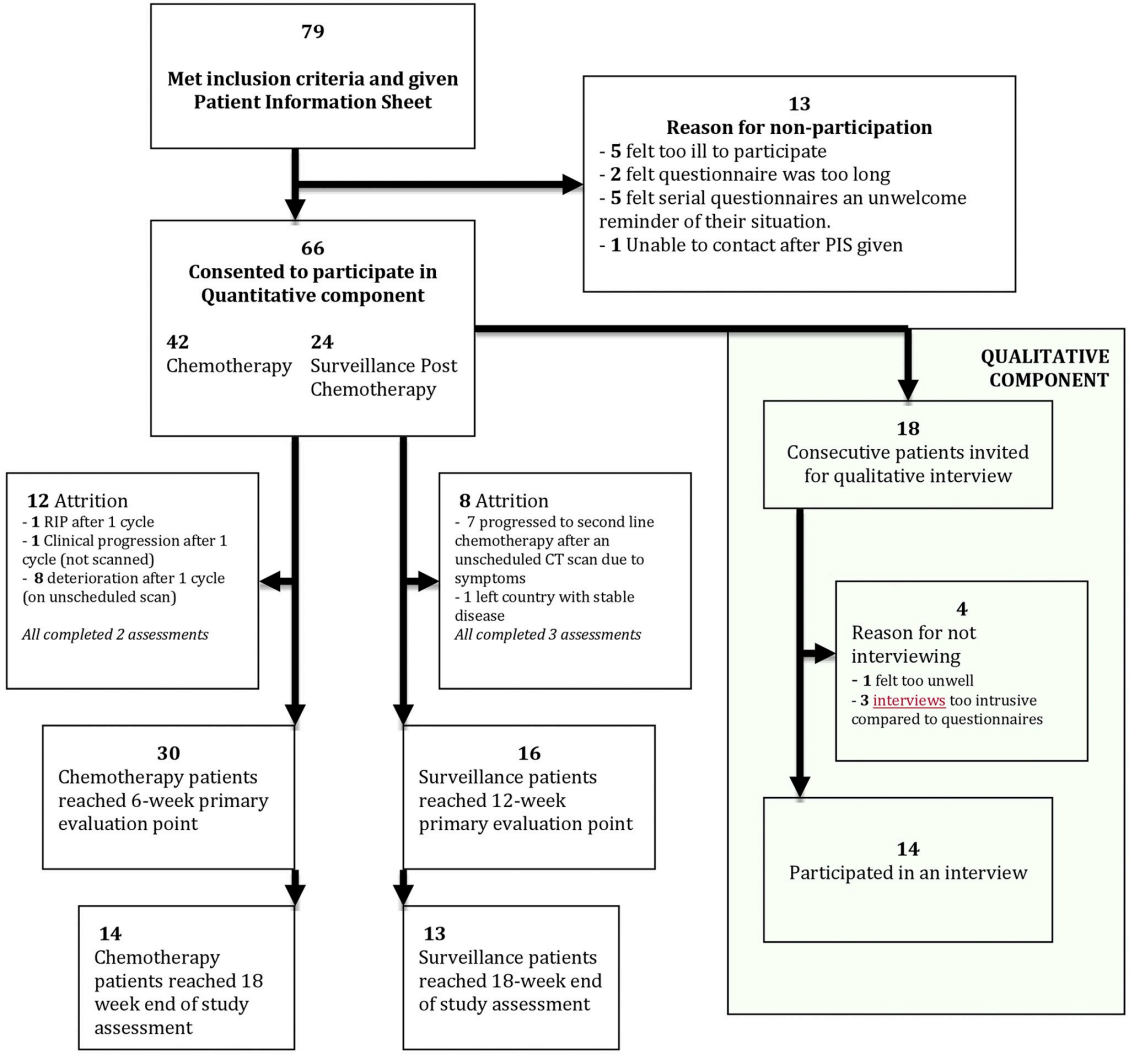
Recruitment and attrition during the mixed methods study.

Baseline quantitative interviews were conducted by NG. Demographic and clinical characteristics were recorded for all participants and the first EORTC QLQ-C30 questionnaire was completed. Participants completed subsequent questionnaires at 2, 6, 12 and 18 weeks after commencing chemotherapy or surveillance (Fig 1). These time points were specifically chosen to correspond with the 3-weekly chemotherapy cycles: the primary evaluation point was after 2-cycles of chemotherapy (after week 6) when the first scan assessing response was routinely performed. The evaluation scan is performed at this time as it is deemed the earliest time a positive radiological response would be detected [26]. However, evaluations were performed before the results of the post 6 weeks treatment scans were known. Those in the surveillance group were approached, recruited and consented a minimum of 6 weeks post-completion of their 3-weekly, 6-cycle course of first line chemotherapy. Questionnaire completion times were identical to the on-treatment group for logistical simplicity but the primary evaluation was conducted at 12 weeks, this being the scheduled frequency of the first ‘off-treatment’ surveillance scans.

The qualitative interviews similarly took place at the same time points for both groups: after 6 weeks (2 chemotherapy cycles) in the chemotherapy group and after 12 weeks in the surveillance group. In both groups, qualitative interviews took place immediately before the scan to ensure the results did not influence participants’ opinions or judgement of their HRQoL.

### The quantitative interviews

We measured HRQoL using the EORTC QLQ-C30 (Version 3.0)[29]. This cancer specific questionnaire was designed and validated to assess HRQoL in cancer populations undergoing treatment. For more common cancers, there are add-on modules assessing specific symptoms and aspects of HRQoL established as important in a specific cancer type (http://groups.eortc.be/qol/). No specific module currently exists for STS so the core questionnaire was used alone. It comprises 30 items across 9 multi-item scales: global health status/ overall HRQoL (GH), physical, role, cognitive, emotional and social function together with fatigue, pain and nausea and vomiting scores. There are single-item measures for dyspnoea, insomnia, anorexia, constipation, diarrhoea and financial impact. Patients provide self-assessment on a numerical rating scale where the higher the score for GH and the functional ability measures represent better i.e. less impaired domains whereas the higher the score for the physical symptom represents increased severity. Linear transformation is used to standardise the raw score to an overall score ranging from 0–100.

### The qualitative interviews

**Fig 2:**
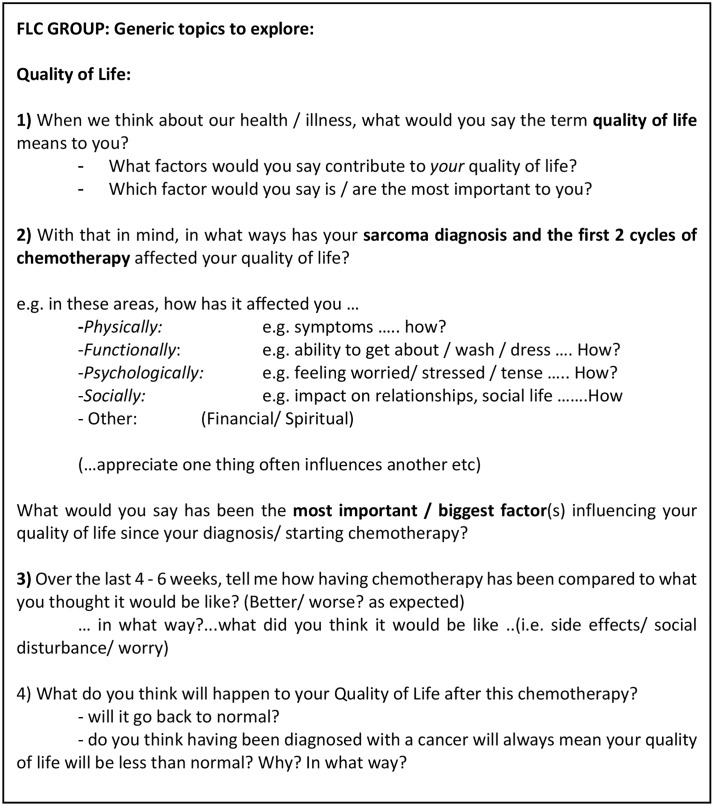
The topic guide used during the qualitative interviews.

Face-to-face semi-structured interviews were offered either to coincide with a future hospital appointment or at the patient’s home at a time convenient for them. Participants could be accompanied if they wished–‘third party’ contributions were welcomed but it was requested only one voice spoke at a time so all contributions were audible. The qualitative interviews followed a topic guide (Fig 2) shaped by comprehensive literature review and a retrospective case note evaluation of deceased patients with STS to examine symptom burden in this patient population and identify symptoms of particular relevance to this patient population[30]. The topic guide was first piloted on four patients and modified after their feedback. Changes included providing patients with greater scope to make the distinction between QoL and HRQoL. All interviews were conducted by NG and digitally recorded; they took place either in participants’ homes or in a quiet room at RM.

### Analysis

#### Quantitative data

Baseline mean EORTC QLQ-C30 data is displayed alongside population reference values (http://groups.eortc.be/qol/) showing comparison of our STS cohort with population control values. The longitudinal data were analysed from a group perspective and a paired t-test applied between baseline and primary evaluation points in the respective groups: baseline to post 2-cycle chemotherapy in the treatment group, baseline to 3-month off-treatment in the surveillance group. Mean differences were considered significant at p<0.05.

#### Missing data

Missing whole questionnaires from patients who dropped out before the primary evaluation points were included in analysis only if they completed a minimum of 2-time points. Their response from the last completed assessment was carried forward to the primary evaluation point for analysis. Any missing data within a returned questionnaire were managed by contacting the participant immediately on its receipt in order to enter missing fields if possible [31].

#### Sample size

Sample size was estimated based on the hypothesis that deterioration in HRQoL score would trigger a supportive referral e.g. to palliative care. This corresponded with a ≥ 10-point reduction in EORTC QLQ-C30 GH-score from baseline to the primary evaluation point. We expected that 20% of those undergoing palliative chemotherapy would have a 10-point reduction in GH score at 6-weeks: we exclude anything less than 5% given there is cost associated with doing the assessments. To detect this effect with 80% power a total of 27 FLC patients would need to be recruited. We used the same sample size for both treatment groups whilst appreciating that the proportion with a 10-point change in GH-score was likely to differ.

Allowing for attrition, the STS-team suggested one-third of chemotherapy patients would not complete 2-cycles, hence we intended to recruit 42 chemotherapy patients to ensure a minimum of 27 evaluable patients. Attrition was predicted to be less in the surveillance group at the 12-week evaluation point so we aimed to recruit 27 patients.

#### Qualitative data

Interviews were transcribed verbatim, anonymised and analysed using the framework approach[32] aided by the qualitative data analysis computer package QSR NVivo (V.10.0). Framework was chosen as it allows step-wise and transparent data analysis permitting both thematic and explanatory analyses. Transcripts were repeatedly read, indexed and relevant sections summarised relating to a specific theme within a framework. This facilitated detailed exploration of the relationship between themes within and across cases. To increase analytical rigour, a subset of transcripts were dual-coded by the research supervisor (JK). A re-iterative process of discussing areas of agreement and disagreement took place between NG and JK to achieve consensus. The analysis was further tested during discussions with colleagues and at project advisory group meetings. Attention was paid to non-confirmatory cases where emerging themes contradicted more common ideas. Excerpts from the transcripts are presented to illustrate themes. Participant anonymity has been preserved throughout by referring to each consecutive participant as ‘participant A’, ‘participant B’ on so on. Where appropriate, participants’ quantitative scores are presented alongside qualitative data.

#### Integration of datasets

Where similar questions relating to HRQoL were assessed both quantitatively and qualitatively, we assessed whether the numbers and themes agreed or disagreed. Where applicable, qualitative data is accompanied by quantitative data to illustrate this[33]. There were some aspects of HRQoL not captured by the EORTC QLQ-C30 which emerged as strong themes in the qualitative interviews: this justified our mixed-methods approach to generate a multi-faceted picture of HRQoL among patients living with STS.

### Ethics

The study received ethical approval (NRES Committee London—Bromley Reference 11/H0803/3). All participants provided written informed consent prior to their involvement in the study.

## Results

Seventy-nine sequential patients met the inclusion criteria of who 66 agreed to participate giving an 84% response rate (Fig 1). Baseline characteristics of all participants are presented in Table 1 and further details of the qualitative sub-group interviewed are found in Table 2.

**Table 1:** Characteristics of those recruited to the quantitative aspect of the study.

Characteristic	Overall advanced sarcoma group (n = 66)	Individual Treatment Group	
		Due to commence First Line Palliative Chemotherapy (n = 42)	Surveillance Post-chemotherapy (n = 24)
**Age at treatment decision**			
Mean ±SD	54.2 ±14	55.9 ±14	53.2+/-13
Median (Range)	55 (18–80)	56 (36–80)	55 (18–74)
**Sex** n(%)			
Male	21 (32)	15 (36)	6 (25)
Female	45 (68)	27 (64)	18 (75)
**Ethnicity** n (%)			
Caucasian	55 (83)	36 (86)	19 (79)
Non-Caucasian	11 (17)	6 (14)	5 (21)
**STS type** n (%)			
Leiomyosarcoma	25 (38)	15 (36)	10 (42)
Pleomorphic	12 (18)	8 (19)	4 (17)
Sarcoma NoS	9 (14)	7 (17)	2 (8)
Liposarcoma	6 (9)	3 (7)	3 (13)
Other *	14 (21)	9 (26)	5 (21)
**Grade** ** n (%)			
2	22 (34)	12 (29)	10 (42)
3	30 (45)	22 (52)	8 (33)
Missing	14 (21)	8 (19)	6 (25)
**Anatomical location** n (%)			
Abdomen / pelvis	35 (53)	23 (55)	12 (50)
Lower limb	11 (17)	8 (19)	3 (13)
Upper limb	2 (3)	1 (2)	1 (4)
Thorax	9 (14)	6 (14)	3 (13)
Retroperitoneum	7 (10)	3 (7)	4 (17)
Unknown	2 (3)	1 (2)	1 (4)
**Median number of metastatic sites** (range)	2 (0–4)	2 (0–4)	2 (0–4)
**Metastatic Site** n (%)			
Lung	44 (67)	27 (64)	17 (71)
Liver	14 (21)	6 (14)	8 (33)
Bone	10 (15)	5 (12)	5 (21)
Soft tissue	29 (44)	19 (45)	10 (42)
LN	12 (18)	7 (17)	5 (21)
Other organ	4 (6)	3 (7)	1 (4)
**Chemotherapy type** n (%)			
Doxorubicin	29 (44)	18 (43)	11 (46)
Ifosfamide/ Doxorubicin	23 (35)	15 (36)	8 (33)
Gemcitabine / Docetaxel	14 (21)	9 (21)	5 (21)
**ECOG Performance Status** -Median (Range)	1 (0–3)	1 (1–3)	1 (0–2)

*Other:

**First Line Chemotherapy:** Synovial 2, Epithelioid 2, endometrial stromal cell 2, epithelioid sarcoma 2, Malignant fibrous histiocytoma 1.

**Surveillance Post-First Line Chemotherapy:** Angiosarcoma 2, Synovial sarcoma 1, Fibrosarcoma 1, epithelioid sarcoma 1

**Low grade STS are rarely ‘chemotherapy-responsive’ and were therefore not included.

**Table 2:** Characteristics of those recruited to the qualitative aspect of the study.

Name	Age	Sex	Ethnicity	STS Histology	New or recurrent advanced disease	Time to interview from advanced diagnosis (Weeks)
**First Line Palliative Chemotherapy**						
**Patient A**	44	F	WB	Leiomyosarcoma	Recurrence	8
**Patient B**	52	F	WB	PeComa	New	8
**Patient C**	56	F	WB	Pleomorphic	Recurrence	7
**Patient D**	60	F	WB	Sarcoma NoS	New	9
**Patient E**	35	M	WB	Synovial	New	6
**Patient F**	38	M	WB	MFH	Recurrence	8
**Patient G**	82	M	WB	Leiomyosarcoma	New	9
**Patient H**	60	M	WB	Epithelioid	Recurrence	8
**Surveillance Post-First Line Chemotherapy**						
**Patient I**	37	F	WB	Epithelioid sarcoma	New	30
**Patient J**	45	F	WB	Leiomyosarcoma	Recurrence	31
**Patient K**	48	F	WB	Liposarcoma	New	30
**Patient L**	56	F	WB	Liposarcoma	Recurrence	29
**Patient M**	53	F	Asian	Leiomyosarcoma	Recurrence	28
**Patient N**	32	M	WO	Fibrosarcoma	Recurrence	32

WB = White British WO = White Other

Of the 42 commencing palliative chemotherapy, 41 completed the 2-week assessment and 30 reached the 6-week (post 2-cycle) primary evaluation point: 14 completed the study (Fig 1). In the surveillance group, 16 of the 24 reached the 3-month primary evaluation point and 13 completed the study. Fourteen patients (8 on chemotherapy, 6 on surveillance) had a face-to-face semi-structured interview. Reasons for non-participation in the study and reasons for participating quantitatively but not wanting an interview are presented in Fig 1.

### Important components of health-related quality of life—Are they captured in EORTC QLQ-C30?

At interview, HRQoL proved a difficult concept for patients to initially understand requiring repeated clarification. All the 14 patients interviewed reported that it was impossible to experience a good QoL without being in ‘good health’. Common ‘factors’ they believed were important in achieving a good HRQoL are presented, together with their frequency, in Table 3. Physical symptoms featured highly and it was acknowledged poorly controlled physical symptoms had a ‘knock-on’ effect on other HRQoL components; for e.g. functional impairment was often a direct consequence of pain that in turn led to loss of independence. The components can be broadly classified into *physical*, *psychological* and *social* domains akin to other HRQoL assessment tools. However, whilst many of the factors highlighted at interview are, in part, assessed in the domains of the EORTC QLQ-C30, some psychological and functional aspects were not (Table 3).

**Table 3:** Participants views on what constitutes having a good HRQoL.

Factor Described	Overall advanced sarcoma group (n = 24)	Individual Treatment Group		Factor Captured in EORTC QLQ-C30
		First Line Chemotherapy (n = 8)	Surveillance Post-FLC (n = 6)	
**Free from pain / symptoms**	12	6	6	Yes
**Time with family and friends**	12	6	6	Yes
**Help with anxiety**	12	6	6	Yes
**Loss of Independence/** **‘Control over life’**	10	6	4	No
**Enjoyment of job**	9	5	4	No
**Outdoor activities**	6	5	1	Yes
**Holidays**	5	4	1	No
**Financial stability**	4	2	2	Yes

Other answers related to pets x2 and religion x2

### Change in overall HRQoL over time

Overall perception of HRQoL over time in the two groups were assessed both quantitatively (GH score of the EORTC QLQ-C30) and qualitatively through an open question during interview as to how, and why, they felt their HRQoL had changed. Table 4 presents mean GH score in both groups and individual scores and perceptions during the interview.

**Table 4:** Triangulation of qualitative and quantitative patient reported perceptions of HRQoL change at respective end points over time.

**Participant**	**Treatment** First Line Chemotherapy	**EORTC QLQ-C30 GH-Score Baseline /100** **(Higher score = better HRQoL)**	**EORTC QLQ-C30 GH-Score post 2-cycles chemo/100** **(Higher score = better HRQoL)**	**Overall HRQoL: Post 2-cycles FLC perception vs. baseline**	**Post** **2-cycle** **(6-week)** **CT scan result**
Patient D	I&Dox	66.6	83.3	Better	R (PR)
Patient B	Dox	33.3	33.3	Worse	P (PD)
Patient F	I&Dox	66.6	33.3	Worse	P (PD)
Patient E	I&Dox	33.3	83.3	Better	R (PR)
Patient C	Dox	75.0	66.6	Worse	P (PD)
Patient G	Dox	100	83.3	Worse	P (PD)
Patient H	I&Dox	83.3	83.3	Worse	R (SD)
Patient A	Dox	100	83.3	Worse	R (SD)
**Average** **(n = 41** **Last Value Carried Forward)**	**/**	**60.4** **(SD 25.0)**	**50.2** **(SD 24.0)**	**-10.2** * (SD 24.1) **t-test: -2.70**	
	**Treatment** Surveillance Post-chemotherapy (FLC received)	**EORTC QLQ-C30 GH Score at Baseline** **(Higher score = better HRQoL)**	**EORTC QLQ-C30 GH Score POM** **(Higher score = better HRQoL)**	**Overall HRQoL:** Post 3-month surveillance perception vs. baseline	**3-month outcome CT-Scan result**
Patient K	I&Dox	58.3	33.3	Worse	P (PD)
Patient J	I&Dox	66.7	66.7	No Change	R (SD)
Patient L	Dox	83.3	83.3	No Change	R (SD)
Patient I	I&Dox	73.4	58.3	No Change	P (PD)
Patient M	Dox	66.7	58.3	No change	P (PD)
Patient N	Dox	66.7	66.7	No change	R (SD)
**Average** **(n = 24 Last Value Carried Forward)**	**/**	**67.4** **(SD 18.2)**	**61.5** **(SD 25.3)**	-5.9** (SD 13.6) **t-test: -1.18**	

* = Significant p = 0.01

** Not significant change

I–ifosfamide, Dox = doxorubicin, R = Responder PR = Partial response SD = Stable disease

P = Progressor PD = progressive disease

Accounts from six of the eight patient participants receiving chemotherapy included perceptions of an overall deterioration in their self-reported HRQoL after 2 cycles. This finding corresponds with the statistically significant reduction in mean GH-score found quantitatively (Table 4). Four of the six participants vocalized how this overall HRQoL deterioration was driven predominantly by an escalation of severity in their physical symptoms, specifically nausea and fatigue:

**‘Patient G**: “Chemo is hard work. It’s so full on. If it’s not the one thing it’s another. The needles, the nausea, the tiredness and having to attend hospital seemingly every week. Quality of life has gone down”

*(Patient G*, *82 years old*, *post 2 cycles single agent doxorubicin)*

Conversely two participants, Patient E and Patient D, who experienced an improvement in their overall HRQoL (both quantitatively and qualitatively) after 2 chemotherapy cycles, explained at interview that this was attributed to an improvement in physical symptoms. Moreover, both were subsequently found to be responding radiologically on scan at the primary evaluation point.

‘**Patient E’:** “I feel my quality of life is better”

**NG:** “Why do you say that?”

**‘Patient E’:** “‘Cause I can breathe and I’m in less pain. All the sickness, the chemo, the trips here are worth it as I’m feeling better: I think you adapt over time as well, you know, become a bit more hardened.”

*(Patient E*, *35 years old*, *post 2 cycles Ifosfamide and doxorubicin)*

**Fig 3:**
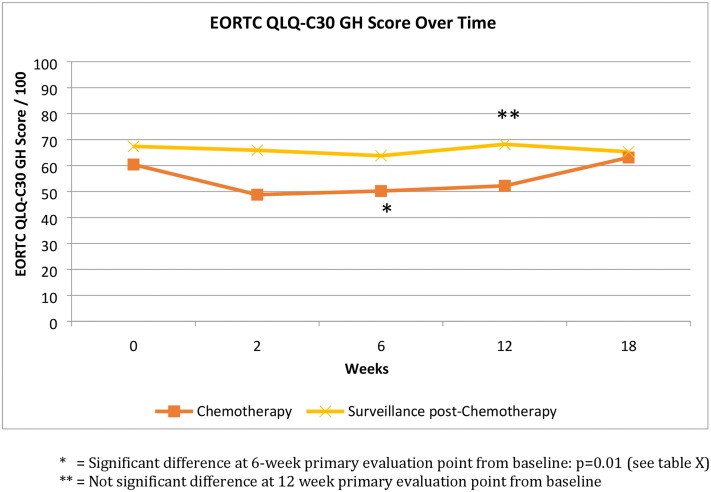
EORTC QLQ-C30 Global Health (GH) score over time in both chemotherapy and surveillance groups. The scores up to the primary evaluation points represent last value carried forward data: n = 42 for the chemotherapy group up to 6 weeks, n = 24 for the surveillance post-chemotherapy group 12-week time point.

In the surveillance group, both quantitative and qualitative findings suggested overall HRQoL scores remained stable over time. However one patient, referred to as Patient K, described deterioration in overall HRQoL largely due to worsening physical symptoms over the 3 months and was found to have progressive disease on subsequent surveillance scan. The mean GH scores from both groups at each time point can be seen in Fig 3.

### Physical symptoms from EORTC QLQ-C30 over time

Compared to population reference values[34], EORTC QLQ-C30 baseline symptom scores were globally worse in both patient groups (Table 5) illustrating the increased prevalence and severity of sarcoma related physical symptoms compared to these symptoms in population control. Specifically, pain, fatigue and sleep disturbance were the three most frequent and severe physical symptoms captured by the EORTC QLQ-C30 in both groups at baseline. In the chemotherapy group, there was a significant deterioration in QLQ-C30 fatigue score (baseline 41.7 to 52.8; change +11.1, t-test +2.51, p<0.05) after 2 cycles. There were, however significant improvements in pain (baseline 41.5 to 32.1; change -9.4, t-test -2.06, p<0.05) and sleep disturbance (baseline 43.1 to 28.5; change -14.6, t-test -3.05, p <0.05) [Table 5].

**Table 5:** Quantitative changes in EORTC QLQ-C30 symptom scores over time in the 2 treatment groups.

**First line Chemotherapy (n = 42)**	**EORTC QLQ-C30 mean Population reference value [34]** **(n = 7802)** **Higher score = worse symptom**	**EORTC QLQ-C30 mean** **Score** **Baseline** **(St Dev)** **Higher score = worse symptom**	**EORTC QLQ-C30 mean score post 2-cycles** **(St Dev)** **Higher score = worse symptom**	**Mean** **Change** **(St Dev)**	**Paired** **t-test** **(Comparing end point to baseline)**	**Statistical Significance** **P-value**
**Fatigue**	**24.1 (24.0)**	**41.7 (31.9)**	**52.8 (28.8)**	**+11.1(28.3)** **[2.2–20.2]**	**+2.51**	**Sig** **(0.016)**
**Nausea/ vomiting**	**3.7 (11.7)**	**15.9 (23.3)**	**21.5 (23.9)**	**+5.6 (23.7)** **[-1.8–13.2]**	**+1.53**	**N/S**
**Pain**	**20.9 (22.8)**	**41.5 (32.5)**	**32.1 (30.8)**	**-9.4 (29.1)** **[-18.5- -0.2]**	**-2.06**	**Sig** **(0.046)**
**Dyspnoea**	**11.8 (22.8)**	**24.4 (29.8)**	**28.5 (30.3)**	**+4.1 (28.1)** **[-4.8–12.9]**	**+0.93**	**N/S**
**Sleep disturbance**	**21.8 (29.7)**	**43.1 (34.4)**	**28.5 (27.4)**	**-14.6 (30.8)** **[-24.3–4.9]**	**-3.05**	**Sig** **(0.004)**
**Appetite loss**	**6.7 (18.3)**	**34.1 (34.6)**	**33.3 (34.2)**	**-0.8 (32.0)** **[-10.9–9.3]**	**-0.16**	**N/S**
**Constipation**	**6.7 (18.4)**	**25.2 (35.6)**	**22.0 (29.4)**	**-3.2 (34.8)** **[-14.2–7.7]**	**-0.60**	**N/S**
**Diarrhoea**	**7.0 (18.0)**	**15.4 (28.0)**	**13.0 (22.2)**	**-2.4 (32.0)** **[-12.5–7.6]**	**-0.49**	**N/S**
**Surveillance Post chemotherapy** **(n = 24)**	**EORTC QLQ-C30 mean Population reference value [34]** **(n = 7802)** **Higher score = worse symptom**	**EORTC QLQ-C30 mean** **Score** **Baseline** **(St Dev)** **Higher score = worse symptom**	**EORTC QLQ-C30 mean score post 2-cycles** **(St Dev)** **Higher score = worse symptom**	**Mean Change** **(St Dev)**	**Paired** **t-test** **(Comparing end point to baseline)**	**Statistical Significance** **P-value**
**Fatigue**	**24.1 (24.0)**	**35.2 (19.0)**	**36.1 (23.7)**	**+0.9 (15.0)** **[-7.3–5.4]**	**+0.38**	**N/S**
**Nausea/ vomiting**	**3.7 (11.7)**	**6.9 (10.9)**	**9.0 (23.7)**	**+2.1 (17.2)** **[-9.4–5.2)**	**+0.80**	**N/S**
**Pain**	**20.9 (22.8)**	**32.6 (32.4)**	**34.7 (32.6)**	**+2.1 (17.9)** **[-9.6–5.5]**	**+0.80**	**N/S**
**Dyspnoea**	**11.8 (22.8)**	**23.6 (25.0)**	**29.2 (30.0)**	**+5.6(30.6)** **[-18.4–7.3]**	**+1.10**	**N/S**
**Sleep disturbance**	**21.8 (29.7)**	**31.9 (37.4)**	**34.7 (37.4)**	**+2.8(29.4)** **[-15.2–9.6}**	**+0.98**	**N/S**
**Appetite loss**	**6.7 (18.3)**	**12.5 (21.6)**	**19.4 (27.6)**	**+6.9(26.0)** **[-17.9–4.0]**	**+1.28**	**N/S**
**Constipation**	**6.7 (18.4)**	**16.7 (22.0)**	**9.7 (18.3)**	**-7.0 (29.4)** **[-5.5–19.4]**	**-1.29**	**N/S**
**Diarrhoea**	**7.0 (18.0)**	**13.8 (25.9)**	**8.3 (17.7)**	**-5.5 (27.2)** **[-5.9–17.0]**	**-1.08**	**N/S**

Patient C and Patient E verbalized both deterioration in fatigue but an improvement in pain, which corresponded with their EORTC QLQ-C30 scores.

**‘Patient C’**: “the tiredness around the diagnosis was bad, the tiredness with this chemo is ‘super-bad’. My “get up and go” has officially got up and gone (laughs)”

*(Patient C*, *56 years old*, *post 2 chemotherapy cycles)*

**‘Patient E’**: “I did get terrible sleep, pain-wise, awful. But it’s changed, it’s getting better. Is that the morphine or the chemotherapy working? I don’t know but it’s definitely getting better. Yep, can definitely do more and sleep better which improves things”

*(Patient E*, *35 years old*, *post 2 cycles Ifosfamide and doxorubicin)*

We observed no significant changes in mean EORTC QLQ-C30 physical symptom scores among those patient participants undergoing surveillance post-chemotherapy.

The presence and control of *physical symptoms* were important to almost all interviewees. Pain, fatigue and the functional impairment associated with these symptoms were frequently vocalised. Extracts from a number of interviews identify key themes. For e.g. Patient K, under surveillance after 6 cycles of chemotherapy, was clear about the negative impact pain and the direct consequences of this symptom were having on her HRQoL.

**‘Patient K’**–”Um .. I suppose I think generally you need your health in order to have a good quality of life. Before diagnosis, I was unwell with pain and that had an impact functionally on me—I couldn’t be a mother, I couldn’t work, I couldn’t enjoy the things I would normally enjoy when I was feeling poorly … so your health is fundamental to you having a good quality of life. I am not good with pain so it ruined my ability to have a good quality of life. Other things generally, um, oh well, my friends, you know, contact with my friends and family er, work if possible, I’ve had to give up work for the time being um but really it’s just that.”

The distinction was also made between STS and treatment-related symptoms. Certain symptoms, for e.g. nausea, were described as ‘cyclical’: in other words bearable since their presence was predictable and would abate with time after treatment. This enabled patients to plan events for times within a cycle when they would be feeling ‘least impaired’.

### EORTC QLQ-C30 domain scores over time

Quantitative functional scores in both groups were lower at baseline compared to population reference values (http://groups.eortc.be/qol/), yet whilst some deterioration over time was seen, no domain changed significantly (at the 5% level) over the course of the study in either group (Table 6). Patient-participants’ qualitative accounts illustrated that living with advanced STS and undergoing chemotherapy or being under radiological surveillance adversely affected their social and psychological wellbeing as time progressed. However these important aspects of their lives were not reflected quantitatively using EORTC QLQ-C30.

**Table 6:** Quantitative changes in EORTC QLQ-C30 Functional scores over time in the 2 treatment groups.

**First line Chemotherapy** (n = 42)	**EORTC QLQ-C30 mean Population reference value [34]** (n = 7802) **Higher score = worse symptom**	**EORTC QLQ-C30 mean** **Score** **Baseline** (St Dev) **Higher score = worse symptom**	**EORTC QLQ-C30 mean score post 2-cycles** (St Dev) **Higher score = worse symptom**	**Mean** **Change** (St Dev)	**Paired** **t-test** (Comparing end point to baseline)	**Statistical Significance** **P-value**
**Functional Scales**						
Physical	89.8 (16.2)	67.2 (26.3)	63.7 (25.9)	-3.4 (17.0) [-8.8–2.0]	-1.29	N/S
Emotional	76.3 (22.8)	68.7 (25.0)	72.8 (25.4)	+ 4.1 (24.7)	+1.05	N/S
Role	84.7 (25.4)	50.4 (41.0)	45.1 (33.4)	-5.2 (32.8) [-15.6–5.1]	-1.03	N/S
Cognitive	86.1 (20.0)	74.8 (23.3)	70.3 (22.2)	-4.5 (23.0) [-11.7–2.8]	-1.25	N/S
Social	87.5 (22.9)	55.7 (35.5)	54.5 (35.0)	-1.2 (24.5) [-9.0–6.5]	-0.32	N/S
Financial	9.5 (23.3)	28.4 (35.4)	27.6 (33.3)	-0.8 (29.3) [-10.1–8.4]	-0.17	N/S
**Surveillance Post chemotherapy** (n = 24)	**EORTC QLQ-C30 mean Population reference value [34]** (n = 7802) **Higher score = worse symptom**	**EORTC QLQ-C30 mean** **Score** **Baseline** (St Dev) **Higher score = worse symptom**	**EORTC QLQ-C30 mean score post 2-cycles** (St Dev) **Higher score = worse symptom**	**Mean Change** (St Dev)	**Paired** **t-test** (Comparing end point to baseline)	**Statistical Significance** **P-value**
**Functional Scales**						
Physical	89.8 (16.2)	67.5 (22.7)	68.1 (21.5)	+0.6 (13.6) [-6.3–5.2]	+0.28	N/S
Emotional	76.3 (22.8)	73.6 (16.6)	69.8 (24.2)	-3.8 (19.5) [-8.8–4.9]	-1.02	N/S
Role	84.7 (25.4)	61.8 (31.3)	61.1 (32.1)	-0.7 (18.0) [-6.9–8.3]	-0.17	N/S
Cognitive	86.1 (20.0)	86.1 (20.1)	85.4 (17.9)	-0.7 (15.1) [-5.7–7.1]	-0.17	N/S
Social	87.5 (22.9)	68.8 (32.3)	70.8 (30.0)	+2.0 (17.2) [-9.3–5.2]	+0.76	N/S
Financial	9.5 (23.3)	23.6 (34.7)	20.8 (32.3)	-2.8 (21.8) [-6.4–12.0]	-0.92	N/S

#### Social domains

The interviews allowed patients to recount components of HRQoL relating to social issues for e.g. the importance of family and friends, more specifically minimizing how their illness or treatment interrupted their family life. A number mentioned how important it was to continue to be effective parents or grandparents where this applied.

Patient F, a 38-year-old, interviewed with his wife after 2 cycles of chemotherapy stated:

**Patient F’: “**It’s a dull ache all the time–when I’m not moving it its ok but not much sets it off. So not only does it stop me working, I can’t even cuddle my kids [upset]…”

His wife explained further

**Wife** “- well no it has, because you haven’t been doing the things that you’ve always done with them, swimming, football, constantly in the garden, our little boy he’s nearly 8, we’re having a bit of a rough time with him at the moment, he’s really playing up, but I think you can see he’s always used to Patient F being so hands on, and he hasn’t been ‘cause of pain and being knackered with this chemo.”

Physical symptoms including pain and fatigue impacted on being able (or wanting) to socialise with others. The presence of such symptoms meant not being able to plan holidays or enjoy leisure pursuits with friends. The side effects of treatment, for e.g. hair loss lead to changes in perception about body image and social confidence.

#### Psychological domains

*Psychological* aspects impacting HRQoL included worry caused by (i) the attribution of symptoms to potential disease activity and (ii) life being ruled by STS and its treatment and (iii) heightened anxiety surrounding a response scan.

## Discussion

To our knowledge, this is the first mixed methods longitudinal study exploring HRQoL among patients living with advanced STS. Our findings identify three areas that warrant further consideration:

First, although many of the factors identified qualitatively as important components of HRQoL are captured in the EORTC QLQ-C30, others such as independence loss and common causes of anxiety are less well assessed. Whilst fixed social and psychological domains are assessed in the EORTC QLQ-C30, the quantitative change overtime did not mirror qualitative descriptions of decline. Second, overall HRQoL deteriorated significantly (both quantitatively and qualitatively) during chemotherapy in the majority. However, there was a trend in the surveillance group (i.e. those who had previously received 6 cycles of chemotherapy and benefitted radiologically [CR/PR or SD]) to have a better overall HRQoL score at baseline than the chemotherapy group at baseline suggesting a correlation with radiological benefit (CR/PR or SD) from chemotherapy and longer-term improvement in HRQoL. Third, the impact (and control) of physical symptoms is central in maintaining HRQoL. Specific symptoms e.g. pain and sleep disturbance, known to be prevalent and severe in those with STS, improved significantly in the chemotherapy group.

### Does chemotherapy in advanced STS really palliate and are we measuring it correctly?

The EORTC QLQ-C30 is a widely used HRQoL questionnaire generic to cancer but has yet to be validated in the STS population. Through patients’ qualitative accounts, we identify that STS had a profound impact on a number of physical, psychological and functional aspects of their lives that were not captured in this commonly used generic tool. Furthermore, our research also provides important evidence that none of the functional scales within the EORTC QLQ-C30 changed over time contrary to the findings from the qualitative accounts of deterioration in social and psychological aspects. This brings into question the tools content validity and responsiveness to change when used in the advanced STS population.

Whilst some of the deficiencies in the EORTC QLQ-C30 identified were ‘psycho-social’ and may apply to other cancer types, our group reported that four of the top 10 most prevalent physical symptoms associated in a cohort of advanced STS patients were absent from the EORTC QLQ-C30[35]. Moreover, physical symptoms including sweats, feeling bloated and coughs were identified using the Memorial symptom assessment scale short form[36]: these were not assessed in the EORTC QLQ-C30. There is therefore an argument to develop either a STS specific HRQoL tool, or an STS specific module to accompany the core EORTC QLQ-C30 questionnaire, a view reiterated by a recent systematic review of the STS literature[37]. Given the heterogeneity of soft tissue sarcomas, this may need to be anatomical or subtype specific. The proactive use of such a tool in the clinical setting may also improve assessment of patients’ clinical needs in the clinical encounter and subsequent focus on their effective management as demonstrated across other cancer sites [38,39].

We identified overall HRQoL deteriorates significantly after 2 cycles of palliative chemotherapy. Whilst attrition made the comparison of both groups over 18 weeks un-reliable, the mean baseline GH scores of those under surveillance following radiological response to chemotherapy were higher than the mean baseline GH scores of those about to commence treatment. This finding suggests HRQoL improves after systemic chemotherapy assuming you have a positive radiological response. Given the lack of convincing data that palliative chemotherapy confers an OS benefit, this finding is clinically promising. Further research is required to confer validation to our observation.

There are few published longitudinal HRQoL data in advanced STS patients undergoing chemotherapy. Poveda [40] reported a lower mean baseline EORTC QLQ-C30 GH-score of 50 in 23 advanced STS patients receiving liposomal doxorubicin as a second line chemotherapy. This lower score was expected given those recruited had progressed on first line chemotherapy with single agent doxorubicin, i.e. these patients were further down the ‘disease trajectory’. After 2 cycles of liposomal doxorubicin, no significant change in mean GH score was observed however data from only 13 patients were reported at this time point.

More recently, HRQoL was evaluated in advanced STS as a secondary end point in the ‘PALETTE trial’. This compared pazopanib with placebo and made use of the EORTC QLQ-C30. The study imputed missing data using linear regression but found no difference between placebo and pazopanib at the 4, 8 or 12-week time points [41].

Previously, our group reported pain, lack of energy and difficulty sleeping were the 3 most prevalent and distressing physical symptoms among a cohort of 113 patients with advanced STS[35]. It is therefore encouraging for these patient groups that the generic EORTC QLQ-C30 assesses these symptoms, also that it identified a significant mean improvement in both pain and sleep disturbance after 2 cycles of chemotherapy.

Establishing the precise aetiology for pain improvement is challenging but is likely multi-factorial. Reasons include: (i) *chemotherapy response–*an exploratory analysis of our data comparing radiological ‘responders (CR/PR or SD)’ with ‘progressors’ identified a trend for improved mean pain score in responders (unpublished data). Studies among patients living with non-small cell lung cancer have observed significant pain improvement in chemotherapy “responders” (CR/PR or SD) but not progressors [42,43,44] (ii) *Increased access to health care professionals* including hospital and community palliative care teams. (iii) ‘*Response shift’* is recognised to affect patients’ perceptions of symptom severity over time that has been demonstrated to show an improvement within patient reported symptom scores over time. [45]

Many patients in this study reported analgesic improvements improved their sleep. This relationship was explored by Grond and colleagues [46] in over 1600 cancer patients where 60% reported insomnia and pain co-existed. Pain severity directly correlated to insomnia. As with other studies, pain, fatigue and insomnia are suggested to form part of a symptom ‘cluster’ [47] [48] where each may cause or influence the outcome of one another.

Fatigue is common amongst cancer patients where its prevalence is reported as between 82–96% [49] among those in receipt of systemic chemotherapy. Qualitative research into CRF suggests it has negative physical and psycho-social HRQoL effects [50]. In this study cancer related fatigue (CRF) increased significantly after two chemotherapy cycles where participants were able to distinguish between disease-related and treatment-related fatigue. Participants reported chemotherapy-related fatigue was predictable and had a cyclical pattern, in that it resolved back to ‘pre-treatment levels’ in the week prior to the next cycle of treatment. Furthermore, our data identified fatigue was physically restrictive; it negatively impacted the ability to work, to function effectively as a parent and pursue leisure and social activities all of which caused considerable distress. Quantitative fatigue scores deteriorated significantly over time with chemotherapy and corresponded with patients’ qualitative accounts. However, although these accounts report extreme functional impairment, this was not represented in the EORTC QLQ-C30 functional domains. This absence of congruity may suggest an issue with the EORTC QLQ-C30 domain scores’ responsiveness to change or that chemotherapy-related fatigue fluctuates during a cycle: we captured data towards the end of a cycle when the symptom was improving.

In terms of the psychological symptoms identified, uncertainty over symptom attribution has been described in advanced cancer[51]. Armstrong found the presence of a new symptom, irrespective of severity, caused concern about its significance [52]. In our study, development of or worsening of a symptom e.g. pain, suggested progressive disease to the participant or implied the need for further treatment in those undergoing surveillance. Conversely, an improvement in analgesia implied a positive radiological response to chemotherapy.

Anxiety surrounding a CT scan, whether scheduled or unscheduled, was a common theme in the qualitative data. Emotional distress is heightened when a CT scan threatened to uncover progressive disease [53]. Whilst many participants described this, there was divergence, given that this qualitative theme was not supported by quantitative emotional function data.

### Limitations

There are a number of limitations in the study that influence the inferences that can be drawn from the results presented. First, this study was inadequately powered to be confident about the generalizability of the study findings. The study findings should therefore be treated as ‘exploratory’ and need further validation in a larger scale study.

The relatively small sample size means this group of patients may not entirely be representative of all sarcoma patients. For example, over 50% of patients had primary tumours in the abdomen or pelvis. This is not typical of all those with STS; if GISTs are excluded the most common primary site for soft tissue sarcomas are the lower limb. The large number of patients with abdominal disease partly reflects the particular interests of the department that has a large referral practice for retroperitoneal and uterine sarcomas. The small sample size also prevented meaningful HRQoL score comparison within the two groups, for example comparing HRQoL scores in the three different regimes of first line chemotherapy received. It could also be argued that to more confidently establish to what extent palliative chemotherapy actually alleviates symptoms associated with highly chemo-sensitive STS subtypes, for example, Ewing sarcoma that are potentially curable despite advanced presentation, they too should have been included in this study.

Second, the surveillance group had, by definition, tolerated and radiologically responded to six chemotherapy cycles and was therefore a selected group in contrast to patients who progress on first line chemotherapy who would either be offered further chemotherapy or end of life care, i.e. would never be in a surveillance situation after first line treatment. Rather than using two separate groups, had time and resources permitted, we would like to have continued to follow the chemotherapy group up to demonstrate changes after chemotherapy with more confidence.

Third, up to the primary evaluation point, we used data from the last completed assessment carried forward to account for whole missing questionnaires. This could be considered to be conservative given that most data were missing due to patients being too unwell to complete the questionnaire. Other, more sophisticated, statistical methods exist for imputing missing data and we recognize using this as a potential limitation [31].

Fourth, STS represents a heterogeneous population both anatomically and in the number of different disease subtypes. Findings would be more generally applicable by limiting the number of subtypes recruited as in Reichardt’s cross-sectional sarcoma HRQoL and health utility study [54].

Last, the qualitative aspect was cross-sectional rather than prospective. Therefore, these data represent a ‘snapshot’ of participants views at a particular time which may have changed if more than one interview had been conducted at a different time point. Henderson and colleagues have shown that many patients with life-limiting conditions, including those approaching the end of life, are in principle agreeable to repeat assessment or interviews [55]. A method to achieve repeat interviews can be achieved through a good initial relationship between interviewer and participant [56]. Longitudinal research has shown this to be feasible during a three year long qualitative study that attempted to explore the processual nature and life changes experienced by 40 women living with breast cancer [57], of whom just over half died by the end of her study.

### Future directions & implications for clinical practice / further research

The study findings, although exploratory, should be fed back to those looking after STS patients. Highlighting patient reported issues might improve the comprehensiveness and effectiveness of the clinical encounter. The study should be repeated on a larger scale i.e. adequately powered, to validate its preliminary findings. Longitudinal interviews are also achievable [56,58] and may improve the assessment of change over time. Given that attrition is a problem in longitudinal studies of advanced diseases, collecting routine data at fixed time points, irrespective of treatment/ disease progression, would provide useful information about disease progression and supportive care referrals. Baseline HRQoL PROM data may even predict progression free survival or treatment response as has been published in other cancer types[59].

## Conclusions

This mixed methods study has identified the limitations of currently available HRQoL tools in relation to this population. Not all aspects of HRQoL considered to be important to those with advanced STS are captured in the current version of EORTC QLQ-C30. Furthermore, functional EORTC QLQ-C30 domains did not change longitudinally in line with qualitative data suggesting poor content validity or response to change in some of the EORTC QLQ-C30 scales. This supports the need for the development and testing of a STS specific HRQoL tool.

Chemotherapy caused overall HRQoL and fatigue to deteriorate but improved both pain and sleep disturbance. Given that baseline overall HRQoL levels returned to above baseline pre-chemotherapy levels in the surveillance group, palliative chemotherapy appeared in part to be achieving its aim. These are clearly findings that warrant further exploration and validation.
